# Non-pharmacological interventions for the prevention of type 2 diabetes mellitus in low and middle-income countries: protocol for a systematic review and meta-analysis of randomized controlled trials

**DOI:** 10.1186/s13643-020-01550-z

**Published:** 2020-12-09

**Authors:** Anupam Sarker, Rina Das, Saraban Ether, K. M. Saif-Ur-Rahman

**Affiliations:** 1 Institute of Epidemiology Disease Control and Research, Dhaka, Bangladesh; 2 Nutrition and Clinical Services Division, icddr,b, Dhaka, Bangladesh; 3 Maternal and Child Health Division, icddr,b, Dhaka, Bangladesh; 4 Health Systems and Population Studies Division, icddr,b, Dhaka, Bangladesh

**Keywords:** Type 2 diabetes, LMICs, Intervention, Non-pharmacological, Systematic review, Meta-analysis

## Abstract

**Introduction:**

The disease burden caused by type 2 diabetes mellitus is a prime public health concern. The prevalence and rate of deaths from diabetes mellitus in low- and middle-income countries (LMICs) are higher than the high-income countries. Increased physical activity and a balanced diet are essential and successful measures to prevent the onset of diabetes mellitus. This systematic review aims to explore the available non-pharmacological approaches for the prevention of type 2 diabetes mellitus in LMICs.

**Methods and analysis:**

Six online databases will be explored to get related randomized controlled trials (RCTs) published in English from inception to September 2020, and two coders will independently screen, identify studies, extract data, and assess the risk of bias in each article. The searched articles will be included by applying specific inclusion and exclusion criteria. Joanna Briggs Institute’s tool for RCTs will be used for appraising the trials critically. Narrative synthesis and pooled effect of the interventions will be demonstrated. A meta-analysis will be conducted using the random-effects model if assumptions are fulfilled.

**Discussion:**

This review is an attempt to explore the available non-pharmacological approaches for the prevention of type 2 diabetes mellitus in LMICs. Findings from the review will highlight effective non-pharmacological measures for the prevention of type 2 diabetes mellitus to guide policy for future strategies.

**Systematic review registration:**

The review protocol has been registered (CRD42020191507).

**Supplementary Information:**

The online version contains supplementary material available at 10.1186/s13643-020-01550-z.

## Background

Diabetes mellitus is considered one of the major non-communicable diseases (NCDs) worldwide. This chronic disease is an effect of either insufficient insulin production by the pancreas or the ineffective utilization of insulin by the body. Over the past few decades, the increased prevalence of diabetes mellitus is a prime public health concern. In 2014, the global prevalence of diabetes mellitus was 422 million [1]. Since 1980, the age-standardized global prevalence of diabetes mellitus has almost been doubled from 4.7 to 8.5%, and in 2012, diabetes mellitus alone was responsible for 1.5 million deaths [2]. It is notified that the prevalence and percentage of death caused by diabetes mellitus are higher in low- and middle-income countries (LMICs) than the high-income countries [2].

Earlier, diabetes mellitus was prevalent among people in affluent countries. But now, almost 80% of people with diabetes mellitus, mainly those who suffer from type 2 diabetes mellitus (T2DM), live in LMICs [3]. Around 336 million people in LMICs are diabetic among which there are a significant number of young adults and children. The total number of diabetic patients is projected to increase to 700 million by 2045 [4]. Additionally, a huge number of people are at risk to be undiagnosed or poorly treated in LMICs [4]. Data from the NCD Risk Factor Collaboration showed the fast-growing trend of the burden of diabetes mellitus in LMICs than in high-income countries [5]. The 9th edition of the IDF Diabetes Atlas has found indistinguishable evidence. According to it, the estimated prevalence of diabetes mellitus among adults aged 20–79 years in high-income countries is 10.4% in 2019 but projected to rise to 11.4% in 2030 and 11.9% in 2045. In the case of middle-income countries, the estimated prevalence is 9.5% in 2019, 10.7% in 2030, and 11.8% in 2045. The estimated prevalence of diabetes mellitus is 4.0% in 2019, 4.3% in 2030, and 4.7% in 2045 in low-income countries [6]. In 2019, among the top 10 countries having the highest number of adults with diabetes mellitus, 8 are from low- or middle-income country groups [6].

Several factors are playing a crucial role to influence the increasing prevalence of diabetes mellitus in LMICs. The most important factors are urbanization, aging, dietary changes, cultural and social changes, physical inactivity, increasing trends in overweight and obesity, changes in diagnostic criteria and screening practices, and better treatment and survival.

There is an escalated predisposition of type 2 diabetes mellitus among South Asians [7] which is comprised of India, Bangladesh, Nepal, Pakistan, Bhutan, Maldives, Sri Lanka, and Afghanistan [8]. A systematic review conducted on the prevalence of diabetes mellitus among South Asian people revealed the increased prevalence of diabetes mellitus over the last two decades [9]. Throughout the urban and peri-urban populations of South Asia, Latin America, and South Africa, the pervasiveness of diabetes mellitus and pre-diabetes is unusually high. Even more alarmingly, in comparison to people in other middle- and low-income countries, South Asians tend to develop diabetes mellitus and pre-diabetes at a younger age and having lower body mass index [10]. The prevalence of diabetes mellitus among the rural population in LMICs was 5.6%, and the distribution is almost similar between men and women. Over the last few decades, diabetes mellitus prevalence has increased keeping an uprising trend, from 1.8% in 1985–1989, 5.0% in 1990–1994, 5.2% in 1995–1999, 6.4% in 2000–2004, and to 8.6% for 2005–2010. The increment is evident to be statistically significant (*p* < 0.001 for secular trend) [11].

Several approaches such as consuming a healthy diet, maintaining normal body weight, regular physical activity, modifying a sedentary lifestyle, and avoiding tobacco use are considered to be effective in preventing type 2 diabetes mellitus or delaying its onset. In the Diabetes Prevention Program (DPP) study, reducing weight was considered as a key element in minimizing the onset of diabetes mellitus. Each kilogram of weight loss is significantly associated with the reduced progression to T2DM and causes a 16% relative risk reduction [12]. In a clinical trial conducted in Da Qing, China, it was found that interventions such as diet or exercise, and a combination of diet and exercise were significantly associated with 31%, 46%, and 42% reductions of risk in developing diabetes, respectively [13]. It is evident from randomized controlled trials that the glucose tolerance level improves with physical activities and reduces the risk of developing T2DM among the high-risk groups. Also, irrespective of body weight status, physical activity reduces the possibility of developing T2DM. Non-pharmacological interventions are effective among people with impaired glucose tolerance in reducing the risk of T2DM, and such interventions are often evident as effective as their pharmacological counterparts [14].

To achieve well-being, the high prevalence of diabetes mellitus which is an increasing threat for public health in LMICs, should be controlled. Lifestyle modification and other non-pharmacological interventions are game-changers in the prevention strategy of T2DM [15]. Multiple non-pharmacological interventions are touted to be effective which needs to be explored for assessing the effectiveness. However, identifying the effective interventions to implement the most efficient prevention program considering the high disease burden and resource constraints of LMICs is essential. The aim of this systematic review is to explore all the available non-pharmacological and lifestyle modification approaches and interventions implemented in LMICs including dietary restriction and supplement, and exercise for the prevention of diabetes mellitus. The effectiveness of different approaches in the prevention and control of T2DM will be compared. Identifying the most effective intervention will guide to recommend the best possible approach(es) to policymakers as the ideal strategy to reduce the burden of T2DM in resource-poor settings in LMICs.

## Methods

The systematic review protocol is consistent with the standard norms of systematic reviews following PRISMA-P (Preferred Reporting Items for Systematic Review and Meta-Analysis Protocol) guidelines [16] and guidelines provided by the Cochrane group [17]. PRISMA-P checklist has been provided in Additional file 1.

### Inclusion criteria

Studies for this review will be included by the following criteria:

- Population: Adult population aged over 18 years regardless of the urban, peri-urban, or rural area from LMICs (as per World Bank 2020 definition) [18] and inclusive of sex and socio-economic groups will be considered for this review. However, diabetic people, pregnant women (for excluding gestational diabetes), and the population living outside of LMICs will be excluded.
- Intervention: Studies that are describing or assessing the impact of available non-pharmacological (e.g., changes in lifestyle, dietary restriction, physical activity, exercise, psychological support, diabetes mellitus self-management approaches) approaches/intervention [19, 20] to prevent type 2 diabetes in LMICs will be included in this review.
- Comparators: Comparators will be the comparison groups which is either the control arm or no intervention group or placebo group (if any).
- Outcomes:
  - *Primary outcomes*: The primary outcome of this review will be to measure the change of incidence of type 2 diabetes mellitus following the non-pharmacological intervention. We will explore the case definitions of T2DM as mentioned in the included articles.
  - *Secondary outcomes*: Secondary outcome will be to find out the effectiveness of available non-pharmacological approaches that are trying to prevent type 2 diabetes mellitus. HbA1c level, weight/BMI, fasting glucose level, 2-h glucose changes from baseline, all-cause mortality, serious adverse health events, patient compliance associated with non-pharmacological approaches, etc. will be the indicators of choice for the secondary outcome.
- Study design: This systematic review will be limited to only randomized controlled trials (RCTs) for portraying the effect of available interventions. Other experimental designs such as non-randomized studies, pretest-posttest design, case-control studies, descriptive studies, prospective comparative cohort studies, and cross-sectional studies will be excluded.

### Exclusion criteria

Studies that are not focusing the World Bank working group’s LMIC list will be excluded. However, if any study includes data from both high-income countries and LMICs, only LMIC data will be considered. Studies will be also excluded if the article is written in other languages than English. Population aged < 18 years of age, people diagnosed as T2DM patients, and those having other health issues will be excluded. Studies that are focusing on pharmacological or surgical intervention or a combination of these interventions with the non-pharmacological intervention will be also excluded from this review. Along with this, any review studies such as systematic reviews, narrative reviews, ongoing RCTs, and trial protocols will be excluded. Other than that, opinion articles, letters, comments, editorials, periodicals, updates, speeches, books, book chapters, book reviews, etc. will be excluded from this review.

### Information sources

According to the research question, a comprehensive search strategy will be developed to search the relevant studies from the following electronic bibliographic databases. These are Medline through PubMed, Embase, the Cochrane Library (Cochrane Central Register of Controlled Trials (CENTRAL)), Web of Science, ClinicalTrials.gov, and ICTRP (International Clinical Trials Registry Platform).

### Search strategy

Through a systematic approach, relevant studies for this review will be searched. Using the keywords and “MeSH” terms according to PICO (population, intervention, comparison, outcome), a detailed comprehensive search strategy will be developed for PubMed which will be adapted for other bibliographical databases in combination with database-specific filters later on. Wild cards and truncations will be used in search terms where necessary.

Table 1 demonstrates the key terms according to PICO that will be used in this review. The comprehensive search strategy prepared by using keywords and “MeSH” terms for Medline is provided in Table 2.

**Table 1:** Key terms used for developing a comprehensive search strategy

Population (P)	Intervention (I)	Outcome (O)	Filter
LMICs “Developing country” Urban Rural “Peri urban”	Exercise “Physical activity” “Nutrition therapy” “Meal plan” “Weight loss” “Lifestyle change” “Lifestyle modification”	“Type 2 diabetes mellitus” T2DM “Diabetes mellitus” Diabetes DM	RCTs “Randomized Controlled trials”

**Table 2:** Comprehensive search strategy

Search strategy: PubMed format	
Sl no.	Search queries
1.	(((((LMICs) OR (“developing countries”[MeSH Terms] OR “developing countries”[All Fields] OR “developing country”[All Fields])) OR (Urban)) OR (Rural))) OR (Peri-Urban)
2.	(((((((((((Exercise[MeSH Terms]))) OR (physical exercise[MeSH Terms])) OR (physical exercises[MeSH Terms])) OR (physical activities[MeSH Terms])) OR (physical activity[MeSH Terms]) ) OR (Exercise)) OR (Physical Exercise)) OR (Physical Activities)
3.	(((((((“nutritional support”[MeSH Terms] OR “nutritional support”[All Fields]) OR “nutritional therapy”[All Fields]) OR “nutrition therapy”[MeSH Terms]) OR “nutrition therapy”[All Fields])
4.	((((Meal Plan) OR “meals”[MeSH Terms] OR “meals”[All Fields]) OR “meal”[All Fields]) AND “Plan”[All Fields])
5.	(((“weight loss”[MeSH Terms] OR “weight loss/analysis”[MeSH Terms] OR “weight loss/diet therapy”[MeSH Terms] OR “weight loss/epidemiology”[MeSH Terms] OR “weight loss/metabolism”[MeSH Terms] OR “weight loss/statistics and numerical data”[MeSH Terms]) OR (weight reduction[MeSH Terms])) OR (body weight change[MeSH Terms])) OR (body weight changes[MeSH Terms])
6.	((((((((lifestyle[MeSH Terms]) OR (lifestyle risk reduction[MeSH Terms])) OR (sedentary lifestyle[MeSH Terms])) OR (life style[MeSH Terms])) OR (“life style/epidemiology”[MeSH Terms] OR “life style/analysis”[MeSH Terms])) OR (Lifestyle changes))) OR (lifestyle modifications) OR“life style”[All Fields]) OR “lifestyle”[All Fields]) OR (“Lifestyle Interventions”[All Fields])
7.	(((“diabetes mellitus”[MeSH Terms] OR “diabetes mellitus, type 2”[MeSH Terms]) OR (“diabetes mellitus, type 2/analysis”[MeSH Terms] OR “diabetes mellitus, type 2/blood”[MeSH Terms] OR “diabetes mellitus, type 2/classification”[MeSH Terms] OR “diabetes mellitus, type 2/complications”[MeSH Terms] OR “diabetes mellitus, type 2/diagnosis”[MeSH Terms] OR “diabetes mellitus, type 2/diet therapy”[MeSH Terms] OR “diabetes mellitus, type 2/drug therapy”[MeSH Terms] OR “diabetes mellitus, type 2/epidemiology”[MeSH Terms] OR “diabetes mellitus, type 2/ethnology”[MeSH Terms] OR “diabetes mellitus, type 2/etiology”[MeSH Terms] OR “diabetes mellitus, type 2/history”[MeSH Terms] OR “diabetes mellitus, type 2/physiopathology”[MeSH Terms] OR “diabetes mellitus, type 2/prevention and control”[MeSH Terms] OR “diabetes mellitus, type 2/statistics and numerical data”[MeSH Terms] OR “diabetes mellitus, type 2/therapy”[MeSH Terms] OR “diabetes mellitus/analysis”[MeSH Terms])) OR (“diabetes mellitus/classification”[MeSH Terms] OR “diabetes mellitus/complications”[MeSH Terms] OR “diabetes mellitus/diagnosis”[MeSH Terms] OR “diabetes mellitus/diet therapy”[MeSH Terms] OR “diabetes mellitus/drug therapy”[MeSH Terms] OR “diabetes mellitus/epidemiology”[MeSH Terms] OR “diabetes mellitus/prevention and control”[MeSH Terms] OR “diabetic cardiomyopathies/metabolism”[MeSH Terms])) OR ((“diabetes”[All Fields] AND “mellitus”[All Fields])) OR “diabetes mellitus”[All Fields]) OR “diabetic”[All Fields]) OR “diabetics”[All Fields]) OR “diabetes”[All Fields] OR “T2DM”[All Fields] OR “DM”[All Fields]
8.	“random allocation”[MeSH Terms] OR (“random”[All Fields] AND “allocation”[All Fields]) OR “random allocation”[All Fields] OR “random”[All Fields] OR “randomization”[All Fields] OR “randomized”[All Fields] OR “randomisation”[All Fields] OR “randomisations”[All Fields] OR “randomise”[All Fields] OR “randomised”[All Fields] OR “randomising”[All Fields] OR “randomizations”[All Fields] OR “randomize”[All Fields] OR “randomizes”[All Fields] OR “randomizing”[All Fields] OR “randomness”[All Fields] OR “randoms”[All Fields] OR “clinical trials as topic”[MeSH Terms] OR “clinical trials as topic”[All Fields] OR “trial”[All Fields] OR “trials”[All Fields]
9.	((clinical trials, randomized [MeSH Terms]) OR (controlled clinical trials, randomized [MeSH Terms])) OR (randomization [MeSH Terms]) OR “RCT”[All Fields] OR “RCTs”[All Fields]
10.	1 AND 2 AND 3 AND 4 AND 5 AND 6 AND 7 AND 8 AND 9
11.	Filters applied: Clinical Trial, Randomized Controlled Trial, Humans.

### Data management

EndNote software will be used to manage the retrieved article from different databases that were found by using the abovementioned comprehensive search strategy. A single EndNote library will contain the studies that will be derived from different databases. After uploading all studies in the library, duplication of the articles will be checked and removed accordingly.

### Study selection

The first screening (title–abstract) will be conducted by two independent reviewers according to the inclusion and exclusion criteria. Thus, eligible studies will be selected and included for full-text review. The screening will be done by using EndNote software. A similar approach will be taken for reviewing full texts by two independent reviewers for final inclusion. If a disagreement arises between reviewers at any stage about inclusion, then it will be resolved through consultation with a third reviewer. Exclusion reasons, multiple publications from the same study, etc. will be reported. PRISMA flow diagram will describe how studies were found and included by applying the customized inclusion and exclusion criteria.

### Assessment of methodological quality

For assessing the methodological rigor of the included studies, “Joanna Briggs Institute (JBI) Critical Appraisal tool for randomized controlled trials” will be used [21]. Two independent reviewers will assess the quality of all included studies, while raised disagreement between the reviewers will be discussed with the third reviewer and resolved by consensus.

The JBI tool contains 13 points to assess the quality of RCTs focusing on selection bias, performance bias, detection bias, attrition bias, appropriate outcome measurement, appropriate study design, and appropriate statistical analysis [21]. A rule of thumb for overall grading has been decided. Among 13 screening questions, if 0–3 “yes” answers were obtained, it will be marked as poor quality. Consecutively, if 4–6 question answers for “yes,” that will be marked as moderate quality, and if more than six “yes” answers are found in a study, that will be ranked as a good quality study. An adequate explanation of assessing the quality will be provided to improve the methodological rigor of the review.

### Data extraction

Data will be extracted by two individual reviewers by using the “JBI Data Extraction form for Experimental Studies.” However, the review team has decided to inquire about some other data such as publication year, study site, country, baseline characteristics of study participants, enrolment and attrition rates, and information for assessing the risk of bias along with the data extracted in accordance with the JBI tool. At the commencement of data extraction, pilot data extraction will be done with a 10% eligible study to check the objectivity of the data extraction tool. In this stage, both reviewers will extract data from the same study to check the consistency and level of a similar understanding of extraction. If any disagreement arises between two reviewers, then it will be resolved through discussion and opinion can be taken from the third reviewer if necessary.

### Data synthesis

In the beginning, an exploration of the available interventions will be conducted and mapped accordingly. A short scoping finding will be demonstrated in terms of the year of publication, the region of the studies, and different preventive interventions. A narrative synthesis focusing on the target population (control and intervention group), intervention description, and outcome will be done from the included studies as narrative synthesis helped to compare the similarities and dissimilarities of relatively homogenous studies and help them arranging together [22]. The measure of the effect size will be presented by relative risk (RR) and odds ratio (OR) with 95% confidence interval (CI) for dichotomous data as mentioned in the included articles. Similarly, for continuous data, we will present mean difference (MD) (with 95% CI) and will consider using the standardized mean difference (SMD) (with 95% CI) in case the same outcomes are measured on different scales. If similar intervention for the prevention of type 2 diabetes, similar participant group, and similar outcome measures could have derived from the included studies, then pooled treatment effect will be estimated through meta-analyses. A random-effects model will be considered assuming the potential heterogeneity between the interventions. However, the *I*^2^ statistic will be conducted to measure the heterogeneity around the studies. Also, if different interventions are found instead of getting several similar interventions, a pooled analysis might not be possible. If all the interventions are different, only a narrative synthesis will be conducted instead of a meta-analysis. If we get several similar interventions along with some standalone interventions, a meta-analysis will be conducted pooling the results of similar intervention and standalone interventions will be described narratively. In addition, a subgroup analysis will be conducted considering participant’s age, gender, ethnicity, available interventions, and settings of the study, as the effect of the intervention might vary because of types of interventions and participant’s characteristics. Aiming of conducting sensitivity analysis has taken, if applicable. In addition to this, a funnel plot will be generated through review manager software (RevMan) for assessing potential publication bias for each study. Egger’s test will be conducted as well to explore the publication bias if assumptions are fulfilled. Covariates, such as ethnicity, sex, and age, will be included in meta-regression models to evaluate the effect of participant characteristics on the effectiveness of the interventions (if available). The certainty of the evidence will be assessed following the Grading of Recommendations, Assessment, Development, and Evaluation (GRADE) approach which considers the risk of bias, the inconsistency of findings, indirectness, and imprecision of findings, and publication bias to grade the evidence. According to the GRADE guidelines, the certainty of the evidence will be rated as high, moderate, low, and very low. However, the rating will be applied to the summary of the evidence and not to the individual studies [23].

## Discussions

It is already evident that type 2 diabetes mellitus is immensely affecting people of diverse age groups around the world. Though the prevalence is increasing both in the economically flourished and LMICs, mostly people from LMICs are experiencing the high growth of this [24]. In addition, type 2 diabetes mellitus creates the burden of other deadly NCDs like cardiovascular disease and renal dysfunction [25, 26]. Therefore, this systematic review is particularly aligned with the sustainable development goals (SDGs) as the SDG target 3.4 aimed for a one third reduction in premature mortality from NCDs by 2030 [27]. As a result, it is a timely demand to identify the available and most efficient and feasible non-pharmacological approach to prevent the onset of type 2 diabetes mellitus in LMICs. This review is aiming to explore all the available non-pharmacological interventions and recommend the most effective intervention which has a significant impact on preventing the onset of type 2 diabetes in LMICs and will provide both practical and research implications for the future. As the burden of T2DM is rapidly growing in the low- and middle-income countries, so are the out-of-the-pocket expenditures for non-communicable diseases [28]. If the non-pharmacological interventions can be found to reduce the incidence and control T2DM effectively, then it will reduce the disease burden and economic impact simultaneously. This review will accumulate the empirical evidence of the available LMIC-based non-pharmacological interventions for preventing the onset of type 2 diabetes mellitus, so it might help the government or donor agencies to take a decision on disbursing the future funding on the most effective approach/intervention in similar settings. Along with this, the decision on scaling up of effective intervention could be taken by responsible authorities by observing the success of the available intervention. This review will also provide the opportunity for further research by revealing the research gap on this issue if there is any. To our knowledge, the only limitation of this protocol is considering English language articles. We might miss the evidence published in Chinese, Latin, and other languages if any.

## Supplementary Information

**Additional file 1.** PRISMA-P checklist.

## Data Availability

All the materials are available upon request.

## Abbreviations

CI: Confidence interval
JBI: Joanna Briggs Institute
LMICs: Low- and middle-income countries
MD: Mean difference
NCD: Non-communicable disease
OR: Odds ratio
PRISMA-P: Preferred Reporting Items for Systematic Review and Meta-Analysis Protocol
PICO: Population, intervention, comparison, outcome
RR: Relative risk
RCT: Randomized controlled trial
T2DM: Type 2 diabetes mellitus
